# Knowledge, attitudes, and practices towards COVID-19 among Chinese residents during the rapid rise period of the COVID-19 outbreak: a quick online cross-sectional survey

**DOI:** 10.7150/ijbs.45221

**Published:** 2020-03-15

**Authors:** Bao-Liang Zhong, Wei Luo, Hai-Mei Li, Qian-Qian Zhang, Xiao-Ge Liu, Wen-Tian Li, Yi Li

**Affiliations:** 1 Department of Psychiatry, Wuhan Mental Health Center, Wuhan, Hubei province, China.; 2 Affiliated Wuhan Mental Health Center, Tongji Medical College of Huazhong University of Science & Technology, Wuhan, Hubei province, China.; 3 Research Center for Psychological and Health Sciences, China University of Geosciences, Wuhan, Hubei province, China.

**Keywords:** Knowledge, Attitude, Practice, COVID-19, China

## Abstract

Unprecedented measures have been adopted to control the rapid spread of the ongoing COVID-19 epidemic in China. People's adherence to control measures is affected by their knowledge, attitudes, and practices (KAP) towards COVID-19. In this study, we investigated Chinese residents' KAP towards COVID-19 during the rapid rise period of the outbreak. An online sample of Chinese residents was successfully recruited via the authors' networks with residents and popular media in Hubei, China. A self-developed online KAP questionnaire was completed by the participants. The knowledge questionnaire consisted of 12 questions regarding the clinical characteristics and prevention of COVID-19. Assessments on residents' attitudes and practices towards COVID-19 included questions on confidence in winning the battle against COVID-19 and wearing masks when going out in recent days. Among the survey completers (n=6910), 65.7% were women, 63.5% held a bachelor degree or above, and 56.2% engaged in mental labor. The overall correct rate of the knowledge questionnaire was 90%. The majority of the respondents (97.1%) had confidence that China can win the battle against COVID-19. Nearly all of the participants (98.0%) wore masks when going out in recent days. In multiple logistic regression analyses, the COVID-19 knowledge score (OR: 0.75-0.90, P<0.001) was significantly associated with a lower likelihood of negative attitudes and preventive practices towards COVID-2019. Most Chinese residents of a relatively high socioeconomic status, in particular women, are knowledgeable about COVID-19, hold optimistic attitudes, and have appropriate practices towards COVID-19. Health education programs aimed at improving COVID-19 knowledge are helpful for Chinese residents to hold optimistic attitudes and maintain appropriate practices. Due to the limited sample representativeness, we must be cautious when generalizing these findings to populations of a low socioeconomic status.

## Introduction

Coronavirus disease 2019 (abbreviated “COVID-19”) is an emerging respiratory disease that is caused by a novel coronavirus and was first detected in December 2019 in Wuhan, China. The disease is highly infectious, and its main clinical symptoms include fever, dry cough, fatigue, myalgia, and dyspnea. In China, 18.5% of the patients with COVID-19 develop to the severe stage, which is characterized by acute respiratory distress syndrome, septic shock, difficult-to-tackle metabolic acidosis, and bleeding and coagulation dysfunction 1,2. Empirical clinical data have shown that the overall case fatality rate of COVID-19 is 2.3% in China, much lower than those of SARS (9.5%), MERS (34.4%), and H7N9 (39.0%) 1-3. The ongoing COVID-19 epidemic has spread very quickly, and by February 15, 2020, the virus had reached 26 countries altogether, resulting in 51,857 laboratory-confirmed infections and 1669 deaths, with nearly all infections and deaths occurring in China 4. In response to this serious situation, the World Health Organization (WHO) declared it a public health emergency of international concern on January 30 and called for collaborative efforts of all countries to prevent the rapid spread of COVID-19 5.

Hubei Province, in particular its capital city Wuhan, has been seriously hit by the COVID-19 epidemic. Some unprecedented measures have been adopted to control the COVID-19 transmission in Hubei and other provinces of China, including the suspension of public transportation, the closing of public spaces, close management of communities, and isolation and care for infected people and suspected cases. Until January 27, government authorities had locked down the whole province of Hubei, and Chinese residents, both inside and outside of Hubei, were also required to just stay at home to avoid contacting with others.

The battle against COVID-19 is still continuing in China. To guarantee the final success, people's adherence to these control measures are essential, which is largely affected by their knowledge, attitudes, and practices (KAP) towards COVID-19 in accordance with KAP theory 6,7. Lessons learned from the SARS outbreak in 2003 suggest that knowledge and attitudes towards infectious diseases are associated with level of panic emotion among the population, which can further complicate attempts to prevent the spread of the disease 8,9.

To facilitate outbreak management of COVID-19 in China, there is an urgent need to understand the public's awareness of COVID-19 at this critical moment. In this study, we investigated the KAP towards COVID-19 of Chinese residents during the rapid rise period of the COVID-19 outbreak.

## Methods

### Participants

This cross-sectional survey was conducted from January 27 to February 1, the week immediately after the lockdown of Hubei Province. Because it was not feasible to do a community-based national sampling survey during this special period, we decided to collect the data online. Relying on the authors' networks with local people living in Hubei, a one-page recruitment poster was posted/reposted to moments and groups of their Wechat (similar to “WhatsApp”) and Weibo (similar to “Twitter”) accounts. The poster was also posted on the websites and official Wechat accounts of several local popular media outlets, including *Yangtze River Daily*, *Wuhan - Listen Online*, and *ChuTian Metropolis Daily*. This poster contained a brief introduction on the background, objective, procedures, voluntary nature of participation, declarations of anonymity and confidentiality, and notes for filling in the questionnaire, as well as the link and quick response (QR) code of the online questionnaire. Persons who were of Chinese nationality, were aged 16 years or more, understood the content of the poster, and agreed to participate in the study were instructed to complete the questionnaire via clicking the link or scanning the QR code. Although the questionnaire was distributed by local residents, we did not constrict our sample to Hubei residents only. Residents of other provinces were also eligible for this survey if they were willing to participate.

The Ethics Committee of Wuhan Mental Health Center approved our study protocol and procedures of informed consent before the formal survey. Participants had to answer a yes-no question to confirm their willingness to participate voluntarily. After confirmation of the question, the participant was directed to complete the self-report questionnaire.

### Measures

The questionnaire consisted of two parts: demographics and KAP. Demographic variables included age, gender, marital status, education, occupation, and place of current residence (Hubei vs. other provinces of China).

According to guidelines for clinical and community management of COVID-19 by the National Health Commission of the People's Republic of China 10,11, a COVID-19 knowledge questionnaire was developed by the authors. The questionnaire had 12 questions (Table 1): 4 regarding clinical presentations (K1-K4), 3 regarding transmission routes (K5-K7), and 5 regarding prevention and control (K8-K12) of COVID-19. These questions were answered on a true/false basis with an additional “I don't know” option. A correct answer was assigned 1 point and an incorrect/unknown answer was assigned 0 points. The total knowledge score ranged from 0 to 12, with a higher score denoting a better knowledge of COVID-19. The Cronbach's alpha coefficient of the knowledge questionnaire was 0.71 in our sample, indicating acceptable internal consistency 12.

Attitudes towards COVID-19 were measured by 2 questions (A1-A2, Table 1) about the agreement on the final control of COVID-19 and the confidence in winning the battle against COVID-19. The assessment of respondents' practices was composed of 2 behaviors (P1-P2, Table 1): going to a crowded place and wearing a mask when going out in recent days.

### Statistical analysis

Frequencies of correct knowledge answers and various attitudes and practices were described. Knowledge scores and attitudes and practices of different persons according to demographic characteristics were compared with independent-samples *t* test, one-way analysis of variance (ANOVA), or Chi-square test as appropriate. Multivariable linear regression analysis using all of the demographic variables as independent variables and knowledge score as the outcome variable was conducted to identify factors associated with knowledge. Similarly, binary logistic regression analyses were used to identify factors associated with attitudes and practices. Factors were selected with a backward stepwise method. Unstandardized regression coefficients (β) and odds ratios (ORs) and their 95% confidence intervals (CIs) were used to quantify the associations between variables and KAP. Data analyses were conducted with SPSS version 17.0. The statistical significance level was set at p < 0.05 (two-sided).

## Results

A total of 6919 participants completed the survey questionnaire. After excluding 9 respondents who reported having been infected with the COVID-19 virus, the final sample consisted of 6910 participants. Among this final sample, the average age was 33.0 years (standard deviation [SD]: 10.7, range: 16-87), 4542 (65.7%) were women, 4387 (63.5%) held a bachelor's degree or above, 3881 (56.2%) engaged in mental labor, and 3810 (55.1%) were Hubei residents. Other demographic characteristics are shown in Table 2.

The correct answer rates of the 12 questions on the COVID-19 knowledge questionnaire were 70.2-98.6% (Table 1). The mean COVID-19 knowledge score was 10.8 (SD: 1.6, range: 0-12), suggesting an overall 90% (10.8/12*100) correct rate on this knowledge test. Knowledge scores significantly differed across genders, age-groups, categories of marital status, education levels, and residence places (P<0.001) (Table 2). Multiple linear regression analysis showed that male gender (vs. female, β: -0.284, P<0.001), age-group of 16-29 years (vs. 30-49 years, β: -0.302, P<0.001), marital status of never-married (vs. married, β: -0.215, P<0.001), education of bachelor's degree or lower (vs. master degree and above, β: -0.191 ~ -1.346, P<0.001), and occupations of unemployment (β: -0.158, P=0.040) and students (β: -0.234, P<0.001) (vs. mental labor) were significantly associated with lower knowledge score (Table 3).

The majority of the respondents agreed that COVID-19 will finally be successfully controlled (90.8%). Rates of reporting “disagree” and “I don't know” were 1.9% and 7.3%, respectively. The attitude towards the final success in controlling COVID-19 significantly differed across genders, education levels, occupation categories, and residence places (P<0.05). In additions, respondents reporting “disagree” and “I don't know” had significantly lower knowledge scores than those reporting “agree” (P<0.001) (Table 4). Multiple logistic regression analysis found that marital status of “others” (vs. married, OR: 2.00, P<0.001) and COVID-19 knowledge score (OR: 0.82, P<0.001) were significantly associated with disagreement on the final success in controlling the disease. Female gender (vs. male, OR: 1.50, P<0.001), age-groups of 16-29 years (OR: 1.76, P=0.013) and 30-49 years (OR: 1.54, P=0.048) (vs. 50+ years), education levels of associate's degree or higher (vs. middle school and below, OR: 1.61~2.23, P≤0.008), occupations of unemployment (OR: 1.86, P=0.001) and students (OR: 0.73, P=0.043) (vs. mental labor), residence place of Hubei (vs. other parts of China, OR: 1.40, P=0.001), and COVID-19 knowledge score (OR: 0.81, P<0.001) were significantly associated with the answer of “I don't know” on A1 (Table 5).

Nearly all of the respondents (97.1%) had confidence that China can win the battle against COVID-19, while 2.9% had no such confidence. The attitude towards confidence of winning significantly differed across categories of marital status and education levels (P<0.05). The COVID-19 knowledge scores were significantly lower in persons without than with confidence of winning (P<0.001) (Table 4). Multiple logistic regression analysis showed that education levels of associate's degree or higher (vs. middle school and below, OR: 3.13-5.04, P<0.001) and COVID-19 knowledge score (OR: 0.75, P<0.001) were significantly associated with no confidence of winning.

The vast majority of the participants had not visited any crowded place (96.4%) and wore masks when going out (98.0%) in recent days. There was still a small portion of the participants who had visited crowded places (3.6%) and had not worn masks when leaving home (2.0%) recently. The rates of these two practices significantly differed across demographic groups (P<0.05), except for the rates of going to a crowded place by residence place (Table 6). Multiple logistic regression analysis showed that male gender (vs. female, OR: 1.37, P=0.019), occupation of “students” (vs. mental labor, OR: 1.54, P=0.007), and COVID-19 knowledge score (OR: 0.90, P<0.001) were significantly associated with going to any crowded place. Male gender (vs. female, OR: 1.37, P=0.019), marital status of “others” (vs. married, OR: 2.97, P=0.003), residing in other parts of China (vs. Hubei, OR: 2.70, P<0.001), and COVID-19 knowledge score (OR: 0.78, P<0.001) were significantly associated with not wearing a mask outside (Table 7).

## Discussion

To the best of our knowledge, this is the first study in China examining the KAP towards COVID-19 among Chinese residents. In this predominantly female and well-educated population, we found an overall correct rate of 90% on the knowledge questionnaire, indicating that most respondents are knowledgeable about COVID-19.

The vast majority of the participants also held an optimistic attitude towards the COVID-19 epidemic: 90.8% believed that COVID-19 will finally be successfully controlled, and 97.1% had confidence that China can win the battle against the virus. Despite this, the practices of Chinese residents were very cautious: nearly all avoided crowded places (96.4%) and wore masks when leaving the home (98.0%) during the rapid rise period of the COVID-19 outbreak. We also analyzed the characteristics of KAP towards COVID-19 and identified some demographic factors associated with KAP; these findings are useful for public health policy-makers and health workers to recognize target populations for COVID-19 prevention and health education.

The finding of a high correct rate of COVID-19 knowledge in Chinese residents was unexpected, because this epidemiological survey was conducted during the very early stage of the epidemic. We consider that this is primarily due to the sample characteristics: 82.4% of the study sample held an associate's degree or higher. Because of the serious situation of the epidemic and the overwhelming news reports on this public health emergency, this population would actively learn knowledge of this infectious disease from various channels of information such as CCTV, the official website of the National Health Commission of China and the Wechat official account of the Wuhan Health Commission. The significant positive association between levels of education and COVID-19 knowledge scores supports this speculation.

During the SARS epidemic, 70.1-88.9% of the Chinese residents believed that SARS can be successfully controlled or prevented, and 94.7-100% had confidence that China can win the battle against SARS 13-15. These figures are similar to our findings on the rates of final success and confidence of winning in the battle against COVID-19. The optimistic attitude of the Chinese residents could be related to the unprecedented COVID-19 control measures such as traffic limits all throughout China and the shutdown of cities and counties of Hubei Province, which enhance people's confidence in winning the battle against the virus. Second, the concerted efforts from across the country also increase Chinese people's confidence to overcome the epidemic, for example, to aid the COVID-19 virus control efforts, many provinces had dispatched thousands of medical workers and provided a large number of medical materials to Wuhan after the outbreak. Third, the good knowledge about COVID-19 among the Chinese residents can also explain this phenomenon, because as shown by results of multiple analyses, higher COVID scores were significantly associated with less likelihood of “disagree” and “I don't know” answers to question A1 and “no” answer to question A2.

Although attitudes towards COVID-19 were optimistic, most residents took precautions to prevent infection by COVID-19: not going to crowded places and wearing masks when going outside. These strict preventive practices could be primarily attributed to the very strict prevention and control measures implemented by local governments such as banning public gatherings. Second, they also could be the result of the residents' good knowledge regarding the high infectivity of the COVID-19 virus, which can be easily transmitted between people via invisible respiratory droplets. Unfortunately, the present study still showed that 3.6% residents went to crowded places and 2.0% did not wear masks when leaving homes recently. These potentially risky behaviors were related to male gender, occupation of “students”, marital status of “others”, residing in other parts of China, and poor COVID-19 knowledge. As suggested by findings from previous studies regarding age and gender patterns of risk-taking behaviors 16-18, men and late adolescents are more likely to engage in risk-taking behaviors. In line with these previous findings, we found significant association between male gender and potentially dangerous practices towards COVID-19 in this study. The significantly higher risk of going to a crowded place among students could be ascribed to their young age. The significantly higher risk of not wearing a mask when leaving homes in non-Hubei vs. Hubei residents may be attributed to the less serious situation of the COVID-19 epidemic in non-Hubei parts of China, resulting non-Hubei residents believing that they have a lower risk of infection with the COVID-19 virus.

It is worth mentioning that higher COVID-19 knowledge scores were found to be significantly associated with a lower likelihood of negative attitudes and potentially dangerous practices towards COVID-19 epidemic in this study. These findings clearly indicate the importance of improving residents' COVID-19 knowledge via health education, which may also result in improvements in their attitudes and practices towards COVID-19. Our findings of the demographic factors associated with KAP towards COVID-19 are generally consistent with previous studies on SARS in 2003 19, 20. These findings further suggest that the health education intervention would be more effective if it targets certain demographic groups, for example, the COVID-19 knowledge may be greatly increased if the health education programs are specifically designed for men and persons with a low level of education.

The strength of this study lies in its large sample recruited during a critical period, the early stage of the COVID-19 outbreak. Nevertheless, compared to the most recent national population statistics of China 21, our sample was obviously over-representative of women, well-educated people, and people engaging in mental labor. Given the significant associations between these demographic variables and KAP towards COVID-19 revealed in this study, we may have overestimated knowledge and rates of preventive practices and underestimated rates of positive attitudes towards COVID-19 of Chinese residents. Considering that educational attainment and occupation are often used as proxy measures of socioeconomic status 22, strictly speaking, our findings can only be generalized to Chinese populations of a relatively high socioeconomic status, particularly women.

Due to limited access to internet and online health information resources, vulnerable populations of Chinese society under the COVID-19 epidemic such as older adults and rural people at grass-root level are more likely to have poor knowledge, negative attitudes, and inappropriate preventive practices towards COVID-19. Therefore, KAP towards COVID-19 of vulnerable populations deserves special research attention in today's China. In addition to the limited sample representativeness, the second limitation of this study is the unstandardized and inadequate assessment of attitudes and practices towards COVID, which should be developed via focus group discussion and in-depth interview and constructed as multi-dimensional measures. Due to the very limited time for developing the questionnaire, both were measured with two simple questions only.

In summary, our findings suggest that Chinese residents of a relatively high level of socioeconomic status, in particular women, have had good knowledge, optimistic attitudes, and appropriate practices towards COVID-19 during the rapid rise period of the COVID-19 outbreak. In addition, good COVID-19 knowledge is associated with optimistic attitudes and appropriate practices towards COVID-19, suggesting that health education programs aimed at improving COVID-19 knowledge are helpful for encouraging an optimistic attitudes and maintaining safe practices. Hopefully, under the combined efforts of Chinese authorities and all Chinese residents, China surely will win the battle against COVID-19 in the near future. Due to the limitation in representativeness of the sample, more studies are warranted to investigate the KAP towards COVID-19 among Chinese residents of a low socioeconomic status.

## Figures and Tables

**Table 1:** Questionnaire of knowledge, attitudes, and practice towards COVID-19

Questions	Options
**Knowledge (correct rate, % of the total sample)**	
K1. The main clinical symptoms of COVID-19 are fever, fatigue, dry cough, and myalgia. (96.4)	True, false, I don't know
K2. Unlike the common cold, stuffy nose, runny nose, and sneezing are less common in persons infected with the COVID-19 virus. (70.2)	True, false, I don't know
K3. There currently is no effective cure for COVID-2019, but early symptomatic and supportive treatment can help most patients recover from the infection. (94.0)	True, false, I don't know
K4. Not all persons with COVID-2019 will develop to severe cases. Only those who are elderly, have chronic illnesses, and are obese are more likely to be severe cases. (73.2)	True, false, I don't know
K5. Eating or contacting wild animals would result in the infection by the COVID-19 virus. (91.4)	True, false, I don't know
K6. Persons with COVID-2019 cannot infect the virus to others when a fever is not present. (89.3)	True, false, I don't know
K7. The COVID-19 virus spreads via respiratory droplets of infected individuals. (97.8)	True, false, I don't know
K8. Ordinary residents can wear general medical masks to prevent the infection by the COVID-19 virus. (73.9)	True, false, I don't know
K9. It is not necessary for children and young adults to take measures to prevent the infection by the COVID-19 virus. (96.6)	True, false, I don't know
K10. To prevent the infection by COVID-19, individuals should avoid going to crowded places such as train stations and avoid taking public transportations. (98.6)	True, false, I don't know
K11. Isolation and treatment of people who are infected with the COVID-19 virus are effective ways to reduce the spread of the virus. (98.2)	True, false, I don't know
K12. People who have contact with someone infected with the COVID-19 virus should be immediately isolated in a proper place. In general, the observation period is 14 days. (97.3)	True, false, I don't know
**Attitudes**	
A1. Do you agree that COVID-19 will finally be successfully controlled?	Agree, disagree, I don't know
A2. Do you have confidence that China can win the battle against the COVID-19 virus?	Yes, no
Practices	
P1. In recent days, have you gone to any crowded place?	Yes, no
P2. In recent days, have you worn a mask when leaving home?	Yes, no

**Table 2:** Demographic characteristics of participants and knowledge score of COVID-19 by demographic variables

Characteristics		Number of participants (%)	Knowledge score (mean ± standard deviation)	t/F	P
Gender	Male	2368 (34.3)	10.5 ± 2.0		
	Female	4542 (65.7)	10.9 ± 1.3	9.301	<0.001
Age-group (years)	16-29	2821(40.8)	10.4 ± 1.9		
	30-49	3574(51.7)	11.1 ± 1.2		
	50+	515(7.5)	10.9 ± 1.3	160.683	<0.001
Marital status	Married	3836(55.5)	11.0 ± 1.2		
	Never-married	2744(39.7)	10.4 ± 1.9		
	Others*	330(33.0)	11.0 ± 1.2	146.72	<0.001
Education	Middle school and below	1217 (17.6)	9.7 ± 2.4		
	Associate's degree	1306 (18.9)	10.8 ± 1.5		
	Bachelor's degree	3043 (44.0)	11.0 ± 1.2		
	Master's degree and above	1344 (19.5)	11.2 ± 1.0	262.000	<0.001
Occupation	Physical labor	1191(17.2)	10.7 ± 1.6		
	Unemployed	451(6.5)	10.6 ± 1.8		
	Students	1387(20.1)	10.1 ± 2.1		
	Mental labor	3881(56.2)	11.1 ± 1.2	138.943	<0.001
Place of current residence	Hubei	3810(55.1)	10.7 ± 1.8		
	Other parts of China	3100(44.9)	10.9 ± 1.3	4.774	<0.001

*“Others” included re-married, co-habiting, separated, divorced, and widowed.

**Table 3:** Results of multiple linear regression on factors associated with poor COVID-19 knowledge

Variable	Coefficient	Standard error	t	P
Gender (male vs. female)	-0.284	0.037	7.591	<0.001
Age-group (16-29 vs.30-49 years)	-0.302	0.057	5.337	<0.001
Marital status (never-married vs. married)	-0.215	0.057	3.771	<0.001
Education (middle school and below vs. master's degree and above)	-1.346	0.060	22.030	<0.001
Education (associate's degree vs. master's degree and above)	-0.410	0.057	7.145	<0.001
Education (bachelor's degree vs. master's degree and above)	-0.191	0.048	3.956	<0.001
Occupation (unemployment vs. mental labor)	-0.158	0.077	2.055	0.040
Occupation (students vs. mental labor)	-0.234	0.058	4.027	<0.001

**Table 4:** Attitudes towards COVID-19 by demographic variables

Characteristics		Attitudes, n (%) or mean (standard deviation)				
		A1: final success in controlling			A2: confidence of winning	
		Agree	Disagree	Don't know	Yes	No
Gender	Male	2185 (92.3)	44 (1.9)	139 (5.9)	2295 (96.9)	73 (3.1)
	Female	4086 (90.0)	89 (2.0)	367 (8.1)**	4418 (97.3)	124 (2.7)
Age-group (years)	16-29	2540 (90.0)	65 (2.3)	216 (7.7)	2736 (97.0)	85 (3.0)
	30-49	3249 (90.9)	63 (1.8)	262 (7.3)	3471 (97.1)	103 (2.9)
	50+	482 (93.6)	5 (1.0)	28 (5.4)	506 (98.3)	9 (1.7)
Marital status	Married	3507 (91.4)	59 (1.5)	270 (7.0)	3744 (97.6)	92 (2.4)
	Never-married	2471 (90.1)	64 (2.3)	209 (7.6)	2650 (96.6)	94 (3.4)
	Others#	293 (88.8)	10 (3.0)	27 (8.2)	319 (96.7)	11 (3.3)*
Education	Middle school and below	1110 (91.2)	38 (3.1)	69 (5.7)	1200 (98.6)	17 (1.4)
	Associate's degree	1185 (90.7)	27 (2.1)	94 (7.2)	1274 (97.5)	32 (2.5)
	Bachelor's degree	2753 (90.5)	50 (1.6)	240 (7.9)	2938 (96.5)	105 (3.5)
	Master's degree and above	1223 (91.0)	18 (1.3)	103 (7.7)**	1301 (96.8)	43 (3.2)**
Occupation	Physical labor	1078 (90.5)	27 (2.3)	86 (7.2)	1154 (96.9)	37 (3.1)
	Unemployed	388 (86.0)	14 (3.1)	49 (10.9)	433 (96.0)	18 (4.0)
	Students	1271 (91.6)	31 (2.2)	85 (6.1)	1361 (98.1)	26 (1.9)
	Mental labor	3534 (91.1)	61 (1.6)	286 (7.4) **	3765 (97.0)	116 (3.0)
Place of current residence	Hubei	3430 (90.0)	73 (1.9)	307 (8.1)	3695 (97.0)	115 (3.0)
	Other parts of China	2841 (91.6)	60 (1.9)	199 (6.4)*	3018 (97.4)	82 (2.6)
COVID-19 knowledge score		10.8 (1.5)	10.0 (2.4)	10.3 (2.1)***	10.8 (1.5)	10.0 (2.5)***

#“Others” included re-married, co-habiting, separated, divorced, and widowed. *P<0.05, **P<.01, ***P<0.001.

**Table 5:** Results of multiple binary logistic regression analysis on factors significantly associated with attitudes towards COVID-19

Variable	OR (95%CI)	P
A1: disagree with final success (vs. agree)		
Marital status (others* vs. married)	2.00 (1.01, 3.96)	0.046
COVID-19 knowledge score	0.82 (0.77, 0.88)	<0.001
A1: unknown about final success (vs. agree)		
Gender (female vs. male)	1.50 (1.21, 1.85)	<0.001
Age-group (16-29 vs. 50+ years)	1.76 (1.13, 2.75)	0.013
Age-group (30-49 vs. 50+ years)	1.54 (1.01, 2.37)	0.048
Education (master's degree and above vs. middle school and below)	2.23 (1.54, 3.23)	<0.001
Education (bachelor's degree vs. middle school and below)	2.00 (1.45, 2.78)	<0.001
Education (associate's degree vs. middle school and below)	1.61 (1.13, 2.28)	0.008
Occupation (unemployment vs. mental labor)	1.86 (1.30, 2.65)	0.001
Occupation (students vs. mental labor)	0.73 (0.53, 0.99)	0.043
Residence place (Hubei vs. other parts of China)	1.40 (1.15, 1.70)	0.001
COVID-19 knowledge score	0.81 (0.77, 0.85)	<0.001
A2: no confidence of winning		
Education (master's degree and above vs. middle school and below)	4.98 (2.64, 9.40)	<0.001
Education (bachelor's degree vs. middle school and below)	5.04 (2.82, 9.03)	<0.001
Education (associate's degree vs. middle school and below)	3.13 (1.66, 5.90)	<0.001
COVID-19 knowledge score	0.75 (0.70, 0.80)	<0.001

*“Others” included re-married, co-habiting, separated, divorced, and widowed.

**Table 6:** Practices towards COVID-19 by demographic variables

Characteristics		Practices, n (%) or mean (standard deviation)			
		P1: going to a crowded place		P2: wearing a mask	
		Yes	No	Yes	No
Gender	Male	109 (4.6)	2259 (95.4)	2296 (97.0)	72 (3.0)
	Female	143 (3.1)	4399 (96.9)**	4476 (98.5)	66 (1.5)***
Age-groups (years)	16-29	123 (4.4)	2698 (95.6)	2723 (96.5)	98 (3.5)
	30-49	110 (3.1)	3464 (96.9)	3541 (99.1)	33 (0.9)
	50+	19 (3.7)	496 (96.3)*	508 (98.6)	7 (1.4)***
Marital status	Married	119 (3.1)	3717 (96.9)	3798 (99.0)	38 (1.0)
	Never-married	119 (4.3)	2625 (95.7)	2654 (96.7)	90 (3.3)
	Others#	14 (4.2)	316 (95.8)*	320 (97.0)	10 (3.0)***
Education	Middle school and below	67 (5.5)	1150 (94.5)	1177 (96.7)	40 (3.3)
	Associate degree	44 (3.4)	1262 (96.6)	1284 (98.3)	22 (1.7)
	Bachelor degree	106 (3.5)	2937 (96.5)	2993 (98.4)	50 (1.6)
	Master degree and above	35 (2.6)	1309 (97.4)**	1318 (98.1)	26 (1.9)**
Occupation	Physical labor	51 (4.3)	1140 (95.7)	1171 (98.3)	20 (1.7)
	Unemployed	15 (3.3)	436 (96.7)	442 (98.0)	9 (2.0)
	Students	71 (5.1)	1316 (94.9)	1334 (96.2)	53 (3.8)
	Mental labor	115 (3.0)	3766 (97.0)**	3825 (98.6)	56 (1.4)***
Place of current residence	Hubei	127 (3.3)	3683 (96.7)	3761 (98.7)	49 (1.3)
	Other parts of China	125 (4.0)	2975 (96.0)	3011 (97.1)	89 (2.9)***
COVID-19 knowledge score		10.3 (2.2)	10.8 (1.5)***	10.8 (1.5)	9.3 (3.2)***

#“Others” included re-married, co-habiting, separated, divorced, and widowed. *P<0.05, **P<.01, ***P<0.001.

**Table 7:** Results of multiple binary logistic regression analysis on factors significantly associated with practices towards COVID-19

Variable	OR (95%CI)	P
P1: going to a crowded place		
Gender (male vs. female)	1.37 (1.05, 1.75)	0.019
Occupation (students vs. mental labor)	1.54 (1.12, 2.11)	0.007
COVID-19 knowledge score	0.90 (0.85, 0.96)	0.001
P2: not wearing a mask		
Gender (male vs. female)	1.89 (1.32, 2.63)	0.001
Marital status (others* vs. married)	2.97 (1.46, 6.08)	0.003
Residence place (other parts of China vs. Hubei)	2.70 (1.85, 4.00)	<0.001
COVID-19 knowledge score	0.78 (0.73, 0.83)	<0.001

*“Others” included re-married, co-habiting, separated, divorced, and widowed.
